# Brought to Light: A Fluorogenic Probe to Monitor Immunosuppressants

**DOI:** 10.1021/acscentsci.4c00665

**Published:** 2024-05-09

**Authors:** Marius Werner, Franziska Thomas

**Affiliations:** Heidelberg University, Institute of Organic Chemistry, Im Neuenheimer Feld 270, 69120 Heidelberg, Germany

**Figure 1:**
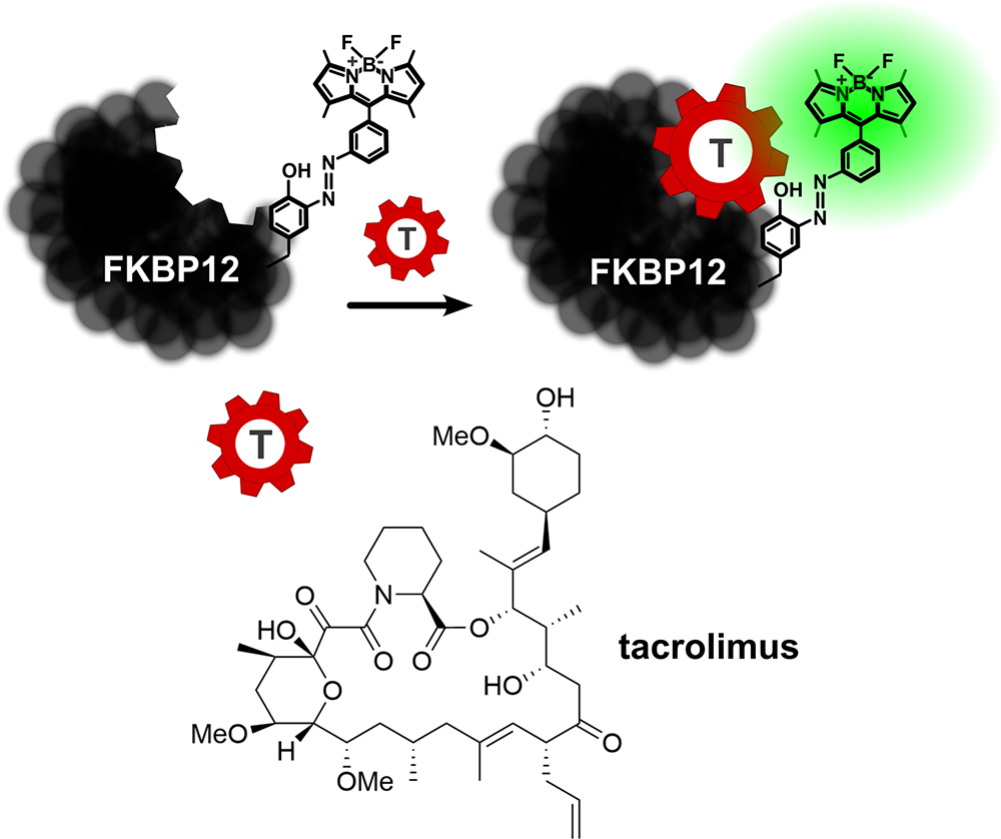
A fluorogenic immunophilin probe for the detection of the immunosuppressant tacrolimus in biosamples. The probe has been developed from the immunophilin FKBP12, to which a BODIPY fluorophore is selectively linked via a tyrosine side chain.

Transplant patients often need to take immunosuppressive drugs to prevent rejection of the transplanted organ. The dosage of these drugs has to be precisely adjusted, which requires monitoring of immunosuppressant levels at the beginning of treatment. Immunoassays are currently the method of choice for monitoring immunosuppressant levels in the blood; however, they cannot monitor in real time, and daily blood sampling can be burdensome for patients. In this issue of *ACS Central Science*, Vendrell, Lavilla, and co-workers describe the development of a fluorogenic probe—a BODIPY-labeled immunophilin—that has the potential to be translated into an approach for monitoring immunosuppressive drug levels in biological samples, such as urine (Figure 1).^1^ This novel approach may open the door to a gentle way of monitoring immunosuppressants in real time, which could even be carried out by the patients themselves, for example, in the form of test strips.

Fluorogenic probes are sensory intelligent molecules that specifically visualize their target by turn-on fluorescence upon binding. In the unbound state, the fluorescence of the probe is negligible, thus sensing with fluorogenic probes is considered to be wash-free. Such probes can therefore be used, for example, in complex biological samples.^2^ Vendrell et al. already have experience in developing fluorogenic peptide probes for *in vivo* labeling. For instance, they showed efficient linker-free coupling of BODIPY to tryptophane.^3−5^ This time they envisaged a fluorogenic protein probe for immunosuppressive drug monitoring using the immunophilins peptidylprolyl isomerase A (PPIA) and FK506-binding protein (FKBP12) as protein receptors that tightly bind immunosuppressive drugs such as tacrolimus. A key aspect of the design was a short linker between the fluorophore and the protein so that the binding of a relatively small ligand, such as the cyclic peptide tacrolimus, would result in the desired turn-on fluorescence.

**Figure 2:**
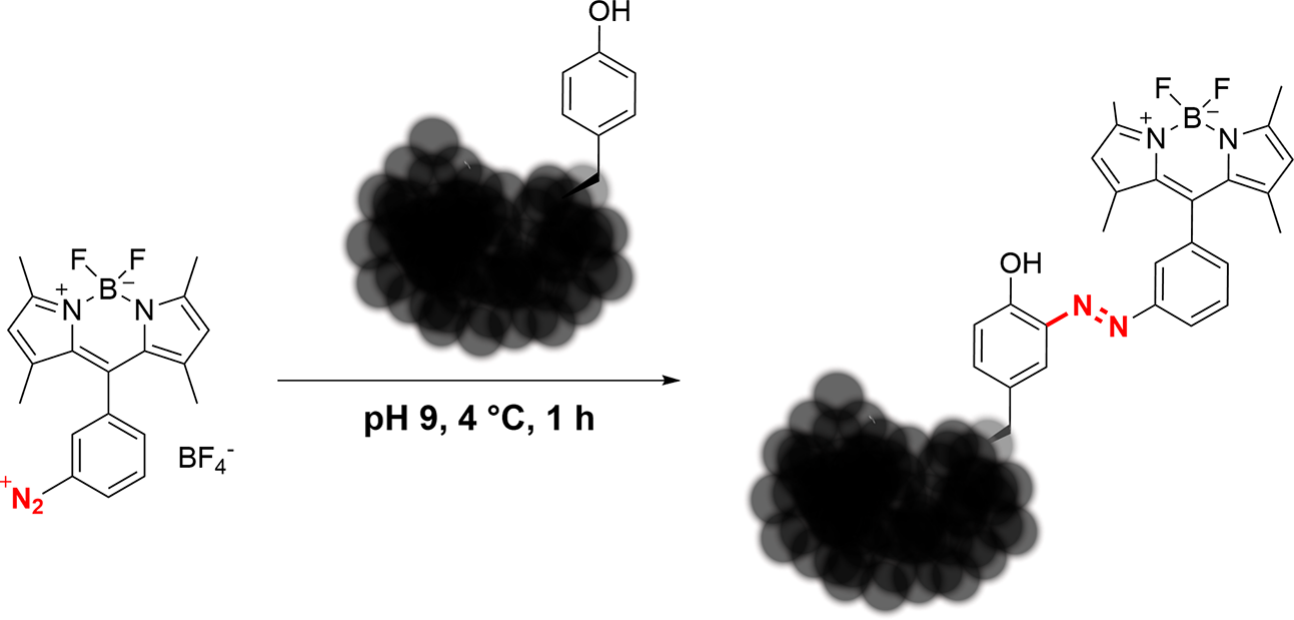
Coupling of the BODIPY diazonium salt to tyrosine side chains. The reaction can be carried out on amino acids, peptides, and proteins and proceeds quickly and under mild conditions.

While peptides can be easily synthesized using solid-phase peptide synthesis and the BODIPY label can be incorporated as a specific amino acid building block, this is not possible for all proteins. In order to achieve the desired design of a fluorogenic immunophilin probe, Vendrell and co-workers developed a new approach for protein modification with BODIPY using site-specific labeling at tyrosine residues. Inspired by the work of the Barbas III group, who previously demonstrated the site-specific modification of tyrosine residues in proteins using aromatic diazonium salts in an azo-coupling reaction, they synthesized the appropriate BODIPY diazonium salt building block and developed a rapid and mild labeling procedure that is applicable at the amino acid, peptide, and protein level (Figure 2).

Modification of proteins at a late stage of synthesis using BODIPY fluorophores in solution is not new.^6^ For instance, methods are available that target the side chains of lysine or cysteine to introduce the BODIPY fluorophore.^7,8^ However, BODIPY fluorophores exhibit self-quenching due to the small Stokes shift, which affects the sensitivity in target recognition. While lysine, a common target for BODIPY labeling, is a common amino acid in natural proteins, tyrosine is rather rare and can often be replaced by phenylalanine without compromising the structural integrity of the protein. The appeal of Vendrell’s method therefore lies in the possibility of avoiding overlabeling, a problem observed when targeting lysine side chains, thus ensuring homogeneously labeled protein species. In their scientific quest to fluorometrically monitor immunosuppressive drug levels, Vendrell and colleagues applied their BODIPY labeling approach to the wild-type immunophilin FKBP12, which contains three tyrosine residues, Y26, Y80, and Y82, with Y82 located near the tacrolimus binding site, and to an FKBP12 variant in which Y26 and Y80 have been modified to phenylalanine (FKBP12 Y26F Y80F). The labeled phenylalanine variant remained correctly folded and retained its binding properties to tacrolimus; in addition, it showed a strong increase in fluorescence emission upon tacrolimus binding. The wild-type FKBP12, on the other hand, was less sensitive to tacrolimus due to the presence of the other tyrosine residues, which highlights the importance of single labeling sites. After optimizing the probe design, Vendrell and co-workers tested the fluorogenic tacrolimus sensor in urine samples from kidney transplant patients who were at risk of organ rejection and therefore treated with tacrolimus. Remarkably, the biosamples from the transplant patients showed higher fluorescence emission when exposed to the immunophilin probe than the control samples from healthy individuals.

Taken together, the research described in this article stands out based on two impressive results: 1) the site-selective labeling of proteins with BODIPY fluorophores on tyrosine side chains using BODIPY diazonium salts and 2) its application to the development of a fluorogenic immunophilin probe that has the potential to be used to monitor immunosuppressant levels in transplant patients. Although the authors did not quantify the amount of immunosuppressants in patients’ urine, they presented a feasibility study that holds great promise for facilitating the adjustment of immunosuppressant doses in transplant patients in the future. If translated into clinical practice, this approach would improve clinical management, minimize the clinical burden, and make monitoring more convenient for patients by avoiding the need for regular blood sampling.
